# Concordance between Coronary Artery Computed Tomography and Invasive Coronary Angiography in a Real-World Population with Suspected Chronic Coronary Syndrome

**DOI:** 10.3390/diagnostics14171905

**Published:** 2024-08-29

**Authors:** Lucia Barbieri, Gabriele Tumminello, Guido Pasero, Carlo Avallone, Andrea D’Errico, Luca Mircoli, Federico Colombo, Cecilia Gobbi, Nello Manuel Bellissimo, Massimiliano Ruscica, Stefano Carugo

**Affiliations:** 1 Department of Cardio-Thoracic-Vascular Diseases, Foundation IRCCS Ca’ Granda Ospedale Maggiore Policlinico, 20122 Milan, Italy; tumminellogabriele@gmail.com (G.T.); luca.mircoli@policlinico.mi.it (L.M.); federico.colombo@policlinico.mi.it (F.C.); cecilia.gobbi@policlinico.mi.it (C.G.); massimiliano.ruscica@unimi.it (M.R.); stefano.carugo@unimi.it (S.C.); 2 Department of Clinical Sciences and Community Health, University of Milan, 20122 Milan, Italy; guido.pasero@unimi.it (G.P.); carlo.avallone@unimi.it (C.A.); nellomanuelbellissimo99@gmail.com (N.M.B.); 3 IRCCS Multimedica, 20138 Milan, Italy; andre.derri@gmail.com; 4 Department of Pharmacological and Biomolecular Sciences “Rodolfo Paoletti”, Università degli Studi di Milano, 20133 Milan, Italy

**Keywords:** coronary artery computed tomography, invasive coronary angiography, chronic coronary syndromes

## Abstract

Background: Coronary computed tomographic angiography (CCTA) is a non-invasive imaging technique that possesses the ability to provide detailed anatomical information about coronary arteries, avoiding unnecessary invasive procedures. Our aim was to assess the ability of CCTA to identify coronary artery disease compared to invasive coronary angiography (ICA) in a real-life setting. Methods: We examined 137 consecutive patients who underwent ICA after CCTA. The latter was conducted in various non-selected centers, and data regarding stenosis were taken from individual reports without further analysis. Results: A total of 60.5% of patients who underwent CCTA were found to have at least one critical stenosis, while the remaining 39.5% underwent ICA due to concurrent clinical or instrumental indications. Among these, 29.5% had angiographically critical pathology, 20.3% underwent a percutaneous coronary intervention (PCI), and 1.8% had coronary artery bypass grafting. Among the 83 patients with positive CCTA results, 34.9% had negative ICA findings. CCTA demonstrated low sensitivity (57.8%) and a positive predictive value of 42.6%. However, it retained high specificity (83.6%) and a negative predictive value of 90.4% for identifying critical stenosis. Among the 18.2% of patients who underwent CCTA without a specific indication, 60% had critical coronary lesions on their ICA and 86.6% of these subsequently underwent a PCI. Conclusions: CCTA performed in non-selective centers has a low concordance with ICA.

## 1. Introduction

Chronic coronary syndrome (CCS) encompasses a spectrum of clinical manifestations resulting from chronic, stable atherosclerotic plaque formation in the coronary arteries. It is a prevalent cardiovascular condition worldwide, significantly contributing to morbidity and mortality [1]. Diagnosing CCS poses several challenges due to the heterogeneous nature of its clinical presentations and the need for accurate risk stratification. Patients with CCS may present with a wide range of symptoms including chest pain, dyspnea, or even silent ischemia. Guidelines suggest using a diagnostic approach based on defining the pre-test probability of disease, estimated from the patient’s clinical presentation, age, and sex [2,3]. Invasive coronary angiography (ICA) is performed in patients with a high clinical likelihood of coronary artery disease (CAD), symptoms unresponsive to medical therapy, angina at low levels of physical exertion, or with warning signs indicating a potential future acute event. Traditionally, ICA has been considered the gold standard for diagnosing CAD, providing detailed information about the location, extent, and severity of the disease [4]. Beyond a luminal stenosis evaluation, ICA can be complemented with intravascular imaging techniques, such as Intravascular Ultrasound (IVUS) and Optical Coherence Tomography (OCT), or a functional analysis like Fractional Flow Reserve (FFR). These tools allow for the assessment of plaque morphology, plaque characteristics, and the hemodynamic significance of coronary lesions [5]. Compared with non-invasive tests, ICA has the advantage of being immediately converted to a therapeutic procedure (e.g., such as a percutaneous coronary intervention—PCI). However, ICA is an invasive procedure with risks, such as bleeding and vascular complications, and should be reserved for selected patients. For patients at a low-to-intermediate risk of CAD, guidelines recommend non-invasive functional or morphological tests [2]. Among these non-invasive imaging techniques, coronary computed tomographic angiography (CCTA) has gained considerable attention for its ability to provide detailed anatomical information about the coronary arteries with high spatial resolution. CCTA allows for the visualization of coronary anatomy, the detection of atherosclerotic plaque burden and characteristics, and the assessment of luminal narrowing. Recent computed tomographic (CT)-associated techniques, such as FFR-CT and CT perfusion, also assess the functional significance of anatomical findings [6]. The main limitation of CCTA is its tendency to overestimate the extent of CAD, particularly when performed in low-volume centers without established expertise or in patients for whom the method is less indicated. Factors such as significant coronary calcifications, irregular heartbeat, obesity, or an inability to cooperate during the examination can adversely affect image clarity [7]. The PROMISE [8] and SCOT-HEART [9] trials found that the use of CCTA was associated with a lower risk of myocardial infarction (MI) compared with the conventional management of CAD patients. These and other studies [10] have strengthened the role of CCTA as a diagnostic tool for CAD, alongside inducible ischemia testing, thus limiting unnecessary invasive procedures and acting as a “gatekeeper” to ICA. Further analysis has investigated the concordance between CCTA and ICA to identify obstructive CAD. A sub-analysis of the ISCHEMIA (The International Study of Comparative Health Effectiveness with Medical and Invasive Approaches) trial [11] showed that in patients with a very high probability of obstructive CAD, CCTA and ICA were concordant in 97.1% of cases for identifying the absence of significant left main (LM) disease and in 92.2% for identifying at least single-vessel CAD. However, their concordance on the extent of the disease based on the number of diseased vessels was moderate (54.5%). The PLATFORM (Prospective LongitudinAl Trial of FFRct: Outcome and Resource IMpacts) study highlighted that in stable, symptomatic patients with suspected CAD, a strategy based on CCTA that is complemented with FFR-CT significantly reduced the rate of planned invasive catheterizations by 60% and lowered the rate of ICA showing no obstructive CAD [12]. This suggests that even in patients with a low-to-intermediate pre-test probability of CAD, a diagnostic strategy that includes systematic CCTA use could reduce unnecessary invasive procedures. Recent studies have confirmed the role of CCTA in low-to-intermediate risk patients and have shown that functional techniques integrated with CCTA can better identify the vessels at risk of causing ischemia, particularly for lesions with stenosis between 40% and 90% [13]. We aimed to assess the ability of CCTA to identify CAD compared with ICA in a real-world population. A secondary goal was to analyze how discrepancies between these two approaches could affect the proper diagnostic and therapeutic care pathways.

## 2. Materials and Methods

We conducted a single-center observational study that involved consecutive patients who underwent coronary angiography following CCTA from September 2019 to February 2024 at the Foundation IRCCS Ca’ Granda Ospedale Maggiore Policlinico (Milan, Italy). Data were collected in a dedicated database, and all patients gave their informed consent for their personal data’s collection. The study was conducted according to GCP, institutional guidelines, national legal requirements, European standards, and the revised Declaration of Helsinki. Hypertension was defined as a systolic blood pressure (BP) > 140 mmHg and/or a diastolic BP > 90 mmHg, or on-treatment with antihypertensive medications. The diagnosis of diabetes was based on a previous history of diabetes treated with or without drugs, fasting glycemia >126 mg/dL, or glycosylated hemoglobin >6.5% [14]. Haemato-chemical examination data were collected upon hospital admission or from the first available tests during hospitalization. Non-fatal MI, major bleeding (defined as Bleeding Academic Research Consortium criteria 3 to 5) [15], ischemic stroke, and unplanned urgent revascularization were recorded as in-hospital adverse events.

### 2.1. CCTA

CCTA was conducted in various non-selected centers, with no limitations on the number of exams performed annually or the types and technical characteristics of the CCTA equipment. The execution of CCTA followed the prescribing physician’s indications according to “real-world” clinical practice. Data on the quantitative assessment of stenosis severity were strictly extracted from individual patient reports without further scan analysis. CCTA was considered positive if it showed a lesion deemed critical by the reporting radiologist or resulted in a luminal stenosis of more than 70%, according to the 2022 CAD-RADS 2.0 classification [16].

### 2.2. ICA

ICA was performed under local anesthesia with a radial approach as the first choice, using the Judkins technique. A quantitative coronary angiography (QCA) analysis was conducted for each patient by two experienced operators using an automated detection system (Siemens Acom Quantcor QCA, Erlangen, Germany). The minimum luminal diameter, reference diameter, stenosis length, and percentage were measured. Stenosis was classified as critical if it was ≥70% in vessels with diameters ≥1.5 mm, intermediate if it was 50–70%, and mild if it was <50% [17]. The decision to perform a coronary angioplasty followed current guidelines and good clinical practices.

Comparison was made between the two methods in terms of their quantitative assessment of stenosis severity, based on the percentages of epicardial coronary vessel stenosis identified through CCTA and subsequent ICA. The analysis was conducted for individual segments and overall, comparing stenosis detection between the two methods. The coronary tree was divided into LM, LAD (including diagonal branches), LCX (including marginal branches and an intermediate branch), and RCA (including posterolateral branches). Secondly, a comparison between the two methods was performed to evaluate individual epicardial branches.

### 2.3. Statistical Analysis

Statistical analysis was performed using SPSS version 23. Continuous variables were expressed as mean ± standard deviation and categorical variables as percentages. Positive and negative predictive values, as well as the sensitivity and specificity, of CCTA compared to coronary angiography, which was used as the gold standard, were calculated. A receiver operating characteristic (ROC) curve was then constructed using dichotomous data, with a fixed threshold based on the previous definition of critical and non-critical, which was obtained from the CCTA and ICA findings of all the investigated segments.

## 3. Results

In total, 137 patients undergoing CCTA and subsequent ICA were analyzed. The population, which was 78% male with a mean age of 67.4 ± 9.8 years, exhibited several cardiovascular risk factors: obesity (18.9%), hypertension (75.9%), dyslipidemia (78%), diabetes (29.2%), a family history of CAD (35%), active smoking (18.2%), and prior smoking (34.3%). The detailed baseline clinical characteristics of the recruited population and their indications for CCTA are provided in Table 1.

In the entire population, 50 out of 137 patients (36.5% of cases) underwent subsequent percutaneous revascularization procedures following coronary angiography. In about half of the cases (47.4%), patients underwent CCTA due to the following symptomatology: exertional angina (29.9%), atypical angina (10.9%), dyspnea (3.6%), prior syncopal episodes (1.5%), or prior episodes of palpitations (1.5%). CCTA was performed in 13.9% of cases for a pre-transplant evaluation (liver or lung transplant), in 13.9% of cases for inducible ischemia on provocative testing (exercise ECG, stress echocardiography, myocardial scintigraphy), and in 6.6% of cases for other indications (such as positive findings on a Holter ECG or abnormalities in regional left ventricular wall motion). Interestingly, in 18.2% of cases, there was no cardiological indication. Of all the CCTAs performed (137 in total), 83 were positive for at least one critical stenosis. The remaining 54 patients underwent coronary angiography due to a concurrent clinical or instrumental indication other than the presence of critical stenosis upon CCTA. Specifically, 24 out of 54 patients showed documented ischemia during a stress test (stress echocardiography or stress test single-photon emission computed tomography), 12 patients were screened for solid organ transplants, and the remaining 18 patients exhibited symptoms of angina or equivalents. When comparing all the analyzed segments, for a total of 548 segments, the non-invasive method had a very low sensitivity (57.8%), a high specificity (83.6%), an extremely low positive predictive value (42.6%), and a very high negative predictive value (90.4%) in identifying critical stenoses. A head-to-head comparison between ICA and CCTA for single epicardial vessels (LM, LAD, LCx, and RCA) showed similar results (Table 2).

**Figure 1:**
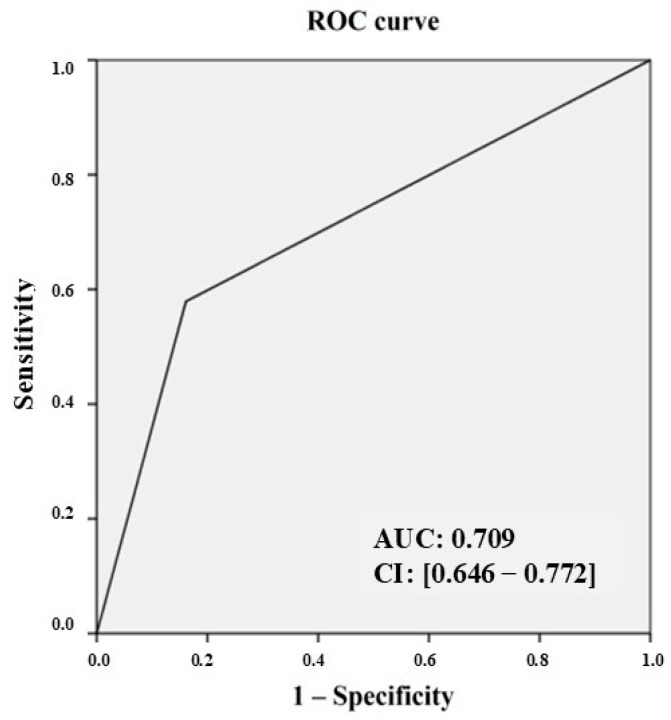
ROC curve developed on CCTA critical segments vs. ICA. AUC = area under the curve; CI = confidence interval.

Considering all 548 segments analyzed by CCTA and the corresponding ICA segments, and using the definition of critical/non-critical, we constructed an ROC curve with an area under the curve of 0.709 and a confidence interval of 0.646–0.772 (Figure 1).

Of the 54 patients undergoing ICA for other clinical–instrumental reasons, despite a negative CCTA for significant stenoses, 14 showed angiographically critical pathology on ICA, mostly involving the RCA, and, in two cases, triple vessel coronary artery disease was found. Of these patients, 11 underwent PCI, 1 underwent CABG, and 2 did not undergo a PCI following negative functional and anatomical evaluation.

### 3.1. Subpopulation with Positive CCTA and Negative ICA Results for Critical Stenosis

Among the patients with positive CCTA results for critical lesions, a total of 29 out of those 83 (34.9%) were identified as having subsequently negative coronary angiography results. Among these patients was the only in-hospital adverse event in the entire population recorded: a post-procedural ischemic stroke. This subpopulation was 69% male, with a mean age of 69.9 ± 9.2 years. Regarding major cardiovascular risk factors, 72.4% had hypertension, 20.7% had diabetes, 79.3% had dyslipidemia, 27.6% had a family history of CAD, and 6.9% had a prior history of coronary artery disease. Additionally, 41.4% were overweight, 13.8% were obese, 20.7% were active smokers, and 34.5% had a history of past smoking. Indications for CCTA were identified within this subpopulation. In nearly half of the cases (44.7%), patients underwent CCTA due to symptomatology that included effort angina (24.1%), non-cardiac chest pain (10.3%), and dyspnea (10.3%). CCTA was performed in 17.2% of cases for pre-transplant evaluation (liver or lung transplant), in 13.8% of cases for inducible ischemia on provocative testing (exercise ECG, stress echocardiography, myocardial scintigraphy), and in 6.9% of cases for other indications (such as positive findings on a Holter ECG or newly detected alterations in regional left ventricular wall motion). The average length of stay for this subpopulation was 2.45 ± 2.37 days, totaling 71 days of hospitalization cumulatively.

### 3.2. Subpopulation Who Underwent CCTA without Specific Indication

A total of 25 patients who underwent CCTA without a specific indication were extracted from the general population (18.2% of the general population). In this subpopulation, 60% tested positive for critical coronary lesions upon angiographic examination (15 patients), and nearly all of them (13 out of 15) subsequently underwent coronary revascularization procedures.

## 4. Discussion

Our study highlighted that in a “real-life” population of patients undergoing CCTA in unselected centers, and based on different clinical –instrumental indications, the confirmation of CAD in a subsequent angiographic examination was often inconsistent. This discrepancy revealed the high specificity and negative predictive value but low sensitivity and positive predictive value of the method. CAD remains one of the leading causes of mortality and morbidity worldwide. Recent European and American guidelines have increasingly emphasized the importance of its timely and effective diagnosis [18]. Hence, non-invasive diagnostic investigations, both anatomical and functional, are recommended as the first-line test to diagnose high-risk CAD patients when a clinical evaluation alone is insufficient. CCTA, due to its intrinsic characteristics, has shown the greatest value in low-risk CAD patients and those with characteristics that lead to higher image quality (younger age, absence of coronary calcifications). Conversely, ICA, although still the gold standard for CAD diagnosis, should be reserved for high-risk patients with persistent symptoms in the context of suspected CCS [2]. The ACCURACY (Assessment by Coronary Computed Tomographic Angiography of Individuals Undergoing Invasive Coronary Angiography) study, which included 230 symptomatic patients with angina undergoing both CCTA and ICA, demonstrated the sensitivity, specificity, positive predictive value, and negative predictive value of CCTA for identifying coronary stenosis higher than 70%, i.e., 94%, 83%, 48%, and 99%, respectively [19]. It is worth noting that all patients were symptomatic for angina in this study, unlike in ours, which included a heterogeneous population both in terms of symptoms and clinical–instrumental indications for performing CCTA. We found a specificity similar to the literature (83.6%) but a lower sensitivity (57.8%) for all analyzed vascular segments [20]. When analyzing single epicardial vessels, both the sensitivity and specificity percentages were even lower. As a confirmation of these values, the area under the curve for the ability of CCTA to match ICA in identifying critical segments was just above 0.7, the minimum acceptable value for a diagnostic test. A sub-analysis of the ISCHEMIA study demonstrated a 97.1% concordance between CCTA and ICA in ruling out left main coronary artery disease and a 92.2% concordance in diagnosing CAD in at least one epicardial vessel within this subgroup [11]. Although the ISCHEMIA study evaluated lesions of 50% or greater, our findings were comparable at a 70% stenosis threshold.

The value of the overall assessment of critical segments for the entire coronary tree was 90.4%, with values ranging between 81.8% and 88.2% for individual segments. Regarding the 83 of 137 patients who underwent coronary angiography due to at least one critical stenosis upon CCTA, 34.9% of patients had no critical stenosis on their ICA. In this group, one peri-procedural stroke occurred; the only in-hospital complication recorded. Additionally, the cumulative hospital stay in this subgroup was 71 days, with an average stay of 2.45 ± 2.37 days per patient, highlighting the potential waste of resources associated with the indiscriminate use of CCTA. Our population was highly heterogeneous both in terms of symptoms and indications for CCTA, and 18.2% did not have any specific indication for undergoing the exam. The SCOT-HEART (Scottish Computed Tomography of the HEART) study suggested that CCTA could increase the frequency and certainty of diagnosing angina due to CAD, which supports the stronger applicability of non-invasive methods as the first diagnostic test in patients with suspected CCS, especially in low-risk patients [9,10,11,12,13,14,15,16,17,18,19,20,21]. On the contrary, in our study population of intermediate to high-risk patients, only the 65.1% with critical lesions on their CCTA also had critical lesions on their coronary angiography. Further, among the 54 patients who underwent ICA without critical lesions on their CCTA, 14 showed angiographically critical disease upon coronary angiography, including two cases of three-vessel disease. Most underestimated lesions on the CCTA involved the right coronary artery, confirming the low positive predictive value of this method at the segmental level among our patients, which is consistent with previous studies. Of these 14 patients, 11 underwent PCI and 1 underwent CABG. These patients were still subjected to ICA despite a negative CCTA for stenosis >70% due to other clinical–instrumental indications, which were primarily exertional angina and inducible ischemia on stress imaging tests, demonstrating the utility of adequate clinical evaluation and the significance of functional tests, especially in a non-selected, real-life population undergoing CCTA. The use of CCTA might increase the number of invasive procedures, especially in the initial clinical management phase of CCS, impacting revascularization procedures with a lower reported long-term myocardial infarction rate [22]. These results align with our observations as most patients were evaluated during their initial diagnostic assessment for suspected CCS. The most alarming finding was that a significant number of CCTA and subsequent ICA tests were performed without a specific reason (18.2%), with the detection of angiographically significant CAD with ICA in 60% of patients and a consequent PCI in almost all patients. The frequency of PCI seemed high compared with the literature, particularly when linked to no underlying clinical indication for CCTA, potentially rendering it a futile procedure. A recent meta-analysis of eight studies involving about 30,000 patients showed that using CCTA as a “gatekeeper” procedure resulted in only a 3% difference in revascularization procedures [23]. The significant increase in CCTA use in daily clinical practice implies that it is not always an effective “gatekeeper” in moderate–high-risk patients, and it may not always be effective for use in primary prevention or for screening asymptomatic patients. Its use has been shown to increase the frequency of invasive procedures and potentially unnecessary PCIs. Our study results highlight the importance of appropriate patient selection, as well as the need for specialized equipment and trained personnel to perform CCTA. This approach ensures that CCTA becomes a suitable and essential diagnostic tool for patients with suspected CCS in the real world. The main limitation of our study is that it is a single-center observational study with a small sample size due to the recruitment period being limited to the last five years. However, the recruitment period was intentionally defined to best evaluate the diagnostic–therapeutic pathway for patients according to updated international guidelines and using the latest generation equipment. The halt of elective activities related to the 2020–2021 global pandemic further reduced the sample size. There was also no selection of centers performing CCTA based on execution volume, thus providing a more realistic evaluation of the concordance between the two diagnostic methods. Furthermore, the indication for performing CCTA and a subsequent ICA was not standardized to capture a real-life snapshot of managing patients with suspected CCS undergoing this type of investigation. This could have led to significant data heterogeneity, which, given the small sample size, prevented specific comparative analyses. Finally, the ICA analysis, considered the gold standard for defining CAD, was performed angiographically by the operators using QCA, following routine clinical practice, and without the assistance of an external core lab.

## 5. Conclusions

Our study showed that in a “real-life” population of patients undergoing CCTA in non-selective centers with various clinical and instrumental indications, the concordance between CCTA and ICA was not high. The discrepancy between real-life data and the scientific literature can be attributed to the latter’s reliance on homogeneous data from high-volume, high-quality technology centers. This suggests that while CCTA is an objective method, its effectiveness depends on the skills of the operator and the center. Additionally, the cumulative hospitalization period and the occurrence of only one peri-procedural complication in patients with positive CCTA and negative ICA highlight the importance of their proper execution and appropriate indication. This is crucial for optimizing economic resources and preventing complications. The high percentage of PCIs in patients without a specific indication for CCTA also suggests that the indiscriminate use of CCTA may lead to overtreatment.

## Figures and Tables

**Table 1:** Clinical baseline characteristics of the population and indications for CCTA.

Clinical Characteristics	
Age (M-SD)	67.4 ± 9.8
Male gender (%)	73
BMI (M–DS)	26.35 ± 4.6
Overweight (%)	43.8
Obesity (%)	13.9
Renal failure (eGFR <60 mL/min/1.73 m^2^) (%)	1.5
Hypertension (%)	75.9
Active smoke (%)	18.2
Prior smoke (%)	34.3
Hypercholesterolemia (%)	73
Family history for CAD (%)	35
Diabetes (%)	29.2
Prior AMI (%)	8
Prior STEMI (%)	5.8
Prior NSTEMI (%)	1.5
Prior ACS (%)	0.7
Prior PCI (%)	13.9
Prior CABG (%)	0
Peripheral vascular disease (%)	5.1
COPD (%)	2.9
Dialysis (%)	0
Creatinine pre	1.00 ± 0.22
Creatinine post	0.97 ± 0.22
Indications for CCTA	
Effort angina (%)	29.9
No indications (%)	18.2
Pre-transplant evaluation (%)	13.9
Positive inducible ischemia test (%)	13.9
Noncardiac chest pain (%)	10.9
Other indications—i.e., positive ECG Holter (%)	6.6
Dyspnea (%)	3.6
Prior syncopal event (%)	1.5
Prior palpitation event (%)	1.5

M = media; SD = standard deviation; AMI = acute myocardial infarction; CABG = Coronary Artery Bypass Graft Surgery; CAD = coronary artery disease; COPD = chronic obstructive pulmonary disease; CCTA = coronary computed tomographic angiography; eGRF = estimated glomerular filtration rate; NSTEMI = Non-ST-Segment Elevation Myocardial Infarction; PCI = percutaneous coronary intervention; ACS = Acute Coronary Syndrome; STEMI = ST-Elevation Myocardial Infarction.

**Table 2:** Comparison between ICA and CCTA according to single epicardial vessels.

	Sn	Sp	PV+	PV−
LM	-	97.7%	-	99.8%
LAD	68.8%	70.6%	53.4%	82.2%
LCx	40.9%	80%	28.1%	87.6%
RCA	55.5%	81.8%	42.8%	88.2%

CCTA = coronary computed tomographic angiography; ICA = invasive coronary angiography; LAD = left anterior descending artery; LCx = left circumflex artery; LM = left main; RCA = right coronary artery; Sn = sensitivity; Sp = specificity; PV = predictive value.

## Data Availability

The original contributions presented in the study are included in the article, further inquiries can be directed to the corresponding author.
